# Probable Rabies Virus Transmission through Organ Transplantation, China, 2015

**DOI:** 10.3201/eid2208.151993

**Published:** 2016-08

**Authors:** Hang Zhou, Wuyang Zhu, Jun Zeng, Jianfeng He, Kai Liu, Yu Li, Shuwu Zhou, Di Mu, Kechun Zhang, Pengcheng Yu, Zhijian Li, Meng Zhang, Xueqiong Chen, Chun Guo, Hongjie Yu

**Affiliations:** Division of Infectious Disease, Key Laboratory of Surveillance and Early-Warning on Infectious Disease, Chinese Center for Disease Control and Prevention, Beijing, China (H. Zhou, Y. Li, D. Mu, C. Guo, H. Yu);; State Key Laboratory of Infectious Disease Prevention and Control, National Institute for Viral Disease Control and Prevention, Chinese Center for Disease Control and Prevention, Beijing (W. Zhu, P. Yu);; Collaborative Innovation Center for Diagnosis and Treatment of Infectious Diseases, Hangzhou, China (W. Zhu, P. Yu);; Guangxi Provincial Center for Disease Control and Prevention, Nanning, China (J. Zeng, S. Zhou);; Guangdong Provincial Center for Disease Control and Prevention, Guangzhou, China (J. He, M. Zhang);; Xiaogan Prefecture Center for Disease Control and prevention, Xiaogan, China (K. Liu); Longhua District Center for Disease Control and Prevention, Shenzhen, China (K. Zhang);; Hezhou Prefecture Center for Disease Control and prevention, Hezhou, China (Z. Li, X. Chen); Fudan University School of Public Health, Key Laboratory of Public Health Safety, Ministry of Education, Shanghai, China (H. Yu)

**Keywords:** rabies, organ transplantation, tissue transplantation, kidney transplant, cornea transplant, infectious encephalitis, China, virus, transmission, transplant recipient, organ donor, transplant-associated transmission

## Abstract

During July 2015, physicians at a hospital in Beijing, China, diagnosed rabies in 2 patients who had each received a kidney from a common organ donor who had died from acute progressive encephalitis of unknown cause. The patients had rabies incubation periods of 42 and 48 days. Altered mental status developed in both patients and progressively worsened to deep coma within 80 days after transplantation; both patients died. Two other transplant recipients received corneas but remained well after receiving timely rabies prophylaxis. An effective regulatory system for testing donors should be implemented to decrease the occurrence of donor-derived infectious diseases. In addition, health education should be improved to enhance public awareness of transplant-associated infectious diseases. Transplant recipients and other persons with exposure to organs or tissues from donors with rabies must be provided consistent health monitoring and follow-up, including rabies postexposure prophylaxis; any remaining organs and tissues must be quarantined and not transplanted.

## Abstract 2

Implementation of an effective regulatory system for testing donors would decrease the occurrence of donor-derived infectious diseases.

In July 2015, physicians at a hospital in Beijing, China, diagnosed rabies in 2 recipients of kidneys from a common organ donor. The donor’s corneas were transplanted into 2 other patients. Rabies virus transmission via nonbite exposures is rare. Thus, the Chinese Center for Disease Control and Prevention (China CDC) initiated an investigation to determine if the virus was transmitted through organ transplantation and to identify and prevent rabies in other transplant recipients and persons who may have been exposed to potentially infectious material.

## Methods

We reviewed medical records for the donor and recipients to determine if the kidney recipients acquired rabies virus through the transplanted organs and to identify other potentially infected recipients of transplants from the same donor. We also interviewed family members of the donor and the deceased kidney recipients.

We used reverse transcription PCR (RT-PCR) targeting the rabies virus nucleoprotein gene (*1*) to extract and amplify RNA from saliva, urine, and sputum samples from kidney transplant recipients. We collected serum samples from the 2 cornea transplant recipients on postexposure prophylaxis (PEP) days 0, 1, 5, 8, 15, and 32. To determine whether an adequate adaptive immune response was produced after vaccination, we measured rabies virus–neutralizing antibody levels in samples by using the rapid fluorescent focus inhibition test (*2*); titers >0.5 IU/mL were considered to provide an adequate level of protection according to World Health Organization standards (*2*).

## Results

### Organ Donor

The donor was a 6-year-old boy who lived in Guangxi Province, an area of China that had the highest number of cumulative reported rabies cases during 2004–2014. During that period, ≈10 rabies cases were reported almost every year in the northeastern part of Guangxi, where the boy resided. On May 13, 2015, the boy had a fever (temperature not recorded) and refused to eat, drink, or sleep (Figure). On May 15th, the boy was sent to a county hospital because the fever and symptoms had not resolved and he showed additional symptoms of extreme irritability, screaming, and slurred speech. The boy refused a physical examination, including measurement of his body temperature. The doctor administered infusion therapy with vitamins B and C, aspirin/glycine , and diazepam, but symptoms persisted.

**Figure:**
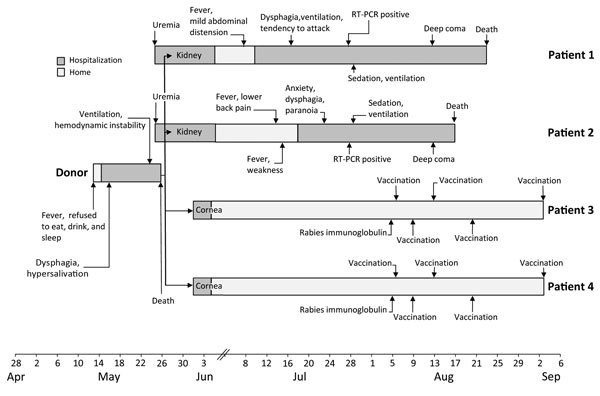
Clinical course of a transplant donor, 2 recipients of kidneys, and 2 recipients of corneas in investigation of probable transplant-associated transmission of rabies virus, China, 2015. RT-PCR, reverse transcription PCR.

On May 16, the boy showed signs of dysphagia and hypersalivation and was moved to another county-level hospital. Doctors presumptively treated the boy with intravenous ribavirin for a possible viral infection, but the boy’s condition continued to deteriorate. Later that day, he was admitted to a prefecture-level hospital with a diagnosis of possible viral encephalitis. Chest and head computed tomography scan images showed pulmonary infection and slightly decreased density of the bilateral temporal lobes. The boy had a leukocyte count of 15.7 × 10^9^ cells/L (reference range 5–12 × 10^9^ cells/L). Cerebral spinal fluid test results showed an opening pressure of 60 drops/min, a transparent and colorless fluid, a protein level of 265.0 mg/L, and a glucose level of 4.70 mmol/L, indicating the probability of viral encephalitis. The boy’s neurologic condition continued to decline, progressing to coma with progressive loss of all physiologic and pathologic reflexes. On May 26, the boy died, and his kidneys and corneas were collected for transplantation; no other organs or tissues were used for transplantation. The boy’s remains were cremated. Before he died, testing was done for HIV, hepatitis B, and syphilis, according to the organ donation law in China; all test results were negative. No autopsy was performed, and no specimens were retained for later testing.

The epidemiologic investigation revealed that the child’s family kept dogs for many years, and the child had had frequent contact with the dogs. However, the boy lived with his elderly grandmother in a different city than that where his parents lived and worked, and the grandmother did not recall any animal bites on the child. In addition, the boy had no travel history, except to attend nursery school, and no pets were kept at the school. The boy had no history of rabies prophylaxis.

### Transplant Recipients

Patient 1 was a 55-year-old man who lived in Hebei Province, China. Because of uremia, he received a kidney allograft transplant on May 27, 2015, in Beijing. Patient 1 had an uncomplicated postoperative course and was discharged home on June 15. During 2 postoperative outpatient visits, he was noted to have a normal recovery. However, on July 8, 42 days after receiving the kidney transplant, he had mild abdominal distension and low-grade fever, and on July 9, limb weakness developed (Figure). He was hospitalized on July 10 for evaluation of fever, fatigue, and worsening muscle soreness. On July 17, he showed symptoms of dysphagia, hearing loss, and incoherent speech and was transferred to the intensive care unit (ICU) and intubated. On July 24, based on the presence of fever, muscle spasms after external stimulation, laryngeal spasms, and muscle atrophy, doctors diagnosed the patient with suspected rabies and transferred him to the infectious disease ICU. On July 28, the diagnosis was confirmed based on rabies-positive RT-PCR results. Patient 1 died on August 23, 46 days after onset of posttransplantion symptoms. Family members reported he had no pets, and they gave no history of animal bites or scratches or of rabies prophylaxis for the patient.

Patient 2 was a 43-year-old man from Liaoning Province, China. On May 27, 2015, he received a kidney allograft transplant in the same hospital and for the same reason (uremia) as patient 1. Patient 2 had an unremarkable postoperative course. He was seen twice as an outpatient and had no unusual findings. On July 14, 48 days after receiving the transplant, he had low back pain, a low-grade fever, and increasing general weakness and leg pain (Figure). He was readmitted to the hospital on July 18 for evaluation of fever and back pain; his speech became incoherent, and he had intermittent fever. On July 21, he had a high fever, anxiety, and paranoia, and on July 22, he had shortness of breath and dysphagia and refused to drink liquids. On July 23, doctors transferred him to an ICU, where seizures subsequently developed. On July 24, the patient was diagnosed with suspected rabies based on the clinical symptoms and was transferred to the infectious disease ICU. On July 28, the diagnosis was confirmed based on rabies-positive RT-PCR results. Patient 2 died on August 17, 34 days after onset of posttransplantation symptoms. Family members reported he had no pets, and they gave no history of animal bites or scratches or of rabies prophylaxis for the patient.

Patient 3 was a 42-year-old man from Guangdong Province, China. The patient had corneal leukoplakia and underwent right eye penetrating keratoplasty and anterior chamber plasty on June 1, 2015. He was discharged on June 12.

Patient 4 was a 62-year-old man from Guangdong Province. He underwent a right eye cornea transplant replacement on June 1 in the same hospital as patient 3. The procedure was done to treat a corneal graft rejection; the operation was successful.

Patients 3 and 4 had no history of chronic disease, bloodborne infection, trauma, blood transfusion, or rabies prophylaxis. They did not have pets and gave no history of animal bites or scratches. After discharge, the patients underwent weekly examinations the first month and monthly examination for the next 2 years. As of February 2, 2016, both had self-reported good health and no discomfort. Corneas were not explanted. Both patients received full rabies PEP beginning on August 5, 2015, immediately following confirmation of corneal transplantation from the donor from Guangxi.

The rabies incubation period for patients 1 and 2 were 42 and 48 days, respectively. Signs and symptoms of altered mental status developed in both patients and progressively worsened to deep coma within 80 days after transplantation (Figure).

We included 290 persons in the epidemiologic investigation of donor and transplant recipient contacts, including family members and healthcare workers in the hospitals. Of the 290 contacts, 233 received PEP. The 57 other evaluated persons did not need PEP because they had not had close contact with the patients or virus.

### Laboratory Findings

#### Patients 1 and 2

Saliva, urine, and sputum samples from patient 1 and saliva and urine samples from patient 2 were positive for rabies virus nucleic acid. Thus, according to diagnostic criteria in China (*3*), both kidney recipients were laboratory-confirmed to be positive for rabies.

#### Patients 3 and 4

Twelve serum samples were collected from the 2 corneal transplant patients. Baseline rabies virus–neutralizing antibody titers on day 0 of PEP were 0.07 IU/mL and 0.02 IU/mL for patients 3 and 4, respectively. However, on PEP day 15, titers were 133.24 IU/mL and 154.78 IU/mL for patients 3 and 4, respectively; on PEP day 32, titers were 168.61 IU/mL and 110.95 IU/mL for patients 3 and 4, respectively. These levels indicate sufficient protection against rabies, according to World Health Organization guidelines (*2*).

## Discussion

Based on these findings, we conclude that rabies in the 2 kidney transplant recipients probably resulted from rabies virus transmitted from the common organ donor. Our findings show that the donor was probably exposed to dogs at home and had symptoms typical of rabies, that neither kidney recipient had a history of exposure to animals with suspected rabies, and that both recipients had posttransplant symptoms of rabies and were PCR-positive for the virus. Further confirmation could not be done because the donor, who was diagnosed with infectious encephalitis, was cremated after organs and tissues were collected for transplantation, and no clinical specimens were kept by the hospital. Thus, we could not confirm rabies in the donor by laboratory methods.

The rabies incubation periods for patients 1 and 2 were 42 and 48 days, respectively, after receiving the kidney transplants, and for both patients, the period from symptom onset to death was >1 month. Similar to findings from previous reports in the transplantation literature, the rabies incubation periods for the 2 kidney recipients were relatively shorter than those for persons infected via the bite route; the long survival periods may also be related to the route of virus transmission (*4*). Rabies did not develop in the cornea recipients before they received PEP, possibly indicating that the virus load in these recipients was lower than that for the kidney recipients (*4*,*5*). We cannot know if rabies would have developed in the 2 cornea transplant recipients if they had not been administered PEP in a timely manner. Rabies also did not develop in other cornea transplant recipients who received timely postexposure rabies PEP or who had transplanted corneas removed (*6*,*7*).

Our study had several limitations. No donor specimens were preserved, an in-depth gene sequencing analysis between donor and recipients could not be conducted, and sequence analyses and a comparison of amplicons from PCR of kidney patients’ saliva were not conducted. In addition, we collected only saliva, urine, and sputum samples from the kidney transplant recipients; cerebrospinal fluid or central nervous system tissues would have been better samples for the laboratory confirmation of rabies.

Rabies has been a notifiable disease in China since 1949. Since then, the highest number of rabies-associated human deaths (>7,000) was reported in 1981 (*8*). The annual incidence has shown a continuous downward trend from 2007 (*9*). However, rabies is believed to be underreported in China because most cases occur in remote rural areas, patients may not be seen by a clinician before death, the proportion of laboratory-confirmed cases is relatively low compared with other causes of illness, and confirmatory laboratory diagnoses is lacking for some cases of infectious encephalitis (*10*). To achieve the goal of rabies elimination, the China CDC is working with the national agricultural departments to improve the national rabies surveillance system, increase testing of suspected human cases of rabies, enhance surveillance and testing of dogs that attack humans, and improve monitoring of rabies PEP clinics.

Based on a regulation issued by the China Ministry of Health in 2006, organs or tissues from HIV-infected or AIDS patients, hepatitis B virus carriers, and persons with other bloodborne diseases, syphilis, or malignant tumors cannot be used for transplantation (*11*). Rabies was not specifically mentioned in the regulation, so it is a challenge to have donors screened for rabies. In some remote or rural areas in China, it is difficult to definitively diagnose the cause of infectious encephalitis, and rabies cases with atypical symptoms might be misdiagnosed. Given that laboratory diagnosis of rabies can only be conducted at prefecture-level and above China CDC laboratories, it is difficult to provide a definitive diagnosis of suspected rabies cases within the short window of time for organ transplantations. Therefore, to reduce infections transmitted from organ donors, we recommend development and implementation of standard questionnaires or tables for use in interviewing relatives of persons who died from infectious encephalitis to identify any rabies exposure history and associated symptoms. Implementation of this questionnaire would prompt regional CDCs to collect specimens from potential donors in time for testing. Cerebrospinal fluid, saliva, and skin biopsies are generally considered as critical samples for antemortem human rabies testing, and brain tissue is considered critical for postmortem confirmation. If time does not permit testing, the presence of infectious encephalitis symptoms in the donor should lead to a determination that organs and tissues are not suitable for transplantation, especially in regions with a high incidence of rabies.

One reason that the rabies cluster reported here was detected is that the 2 kidney recipients were hospitalized in the same facility. If only 1 rabies case is diagnosed in a transplant recipient at a facility, it might be difficult to identify the organ donor as the infectious source.

Other infectious pathogens can also be transmitted during transplantation (*12*–*15*), and the cause of encephalitis among solid organ transplant recipients can be multifactorial. Other infectious agents associated with encephalitis, including West Nile virus, lymphocytic choriomeningitis virus, and *Balamuthia mandrillaris* amebae, have been reported in clusters of solid organ transplant recipients (*16*). The occurrence of donor-derived infectious diseases in transplant recipients can be decreased by strengthening donor assessment and evaluation and by implementing an effective regulatory system for testing donors. In addition, health education should be improved to enhance public awareness of possible transplant-associated infectious diseases. If it is determined that organs or tissues from a donor with rabies have been transplanted, the transplant recipients and other exposed persons who are at risk must receive consistent health monitoring and follow-up, including rabies PEP, and any remaining organs and tissues must be quarantined and not transplanted.
